# Lactobacillus rhamnosus PB01 (DSM 14870) supplementation affects markers of sperm kinematic parameters in a diet-induced obesity mice model

**DOI:** 10.1371/journal.pone.0185964

**Published:** 2017-10-10

**Authors:** Fereshteh Dardmeh, Hiva Alipour, Parisa Gazerani, Gerhard van der Horst, Erik Brandsborg, Hans Ingolf Nielsen

**Affiliations:** 1 Biomedicine Group, Department of Health Science and Technology, Faculty of Medicine, Aalborg University, Aalborg, Denmark; 2 SMI®, Department of Health Science and Technology, Faculty of Medicine, Aalborg University, Aalborg, Denmark; 3 Department of Medical Bioscience, University of the Western Cape, Bellville, South Africa; 4 Bifodan A/S, Hundested, Denmark; University of Melbourne, AUSTRALIA

## Abstract

Probiotics have been proposed as alternatives to pharmacological products in several medical conditions including the modulation of obesity, which is frequently associated with poor semen quality. However, effects of probiotics on male fertility have been less investigated. This study assessed the effect of *Lactobacillus rhamnosus* PB01 (DSM-14870) on sperm kinematic parameters in Normal-weight (NW) and diet-induced obese (DIO) models. NW and DIO C57BL/6NTac mice were divided into two subgroups with or without a single daily dose (1x10^9^CFU) of *L*. *rhamnosus* for four weeks. Sperm motility and kinematics together with blood lipid profiles and reproductive hormone levels were assessed using the sperm class analyzer system. Probiotic supplementation increased serum testosterone, LH and FSH levels in both NW and DIO groups resulting in significantly (P<0.05) higher velocity (VSL, VCL and VAP) and percentages of progressively motile sperm and significantly lower percentages of immotile sperm. Other kinematic parameters (Lin, STR, ALH and BCF) were also increased in both probiotic supplemented DIO and NW groups at the 10% level of significance. Probiotic supplemented DIO mice demonstrated significantly higher percentages of progressively motile sperm versus DIO controls. This study demonstrated the potential of *L*. *rhamnosus PB01* as a regulatory agent with positive effects on weight loss and reproductive-hormones, significantly improving sperm motility and kinematic parameters in male DIO models.

## Introduction

Infertility represents a global problem and is defined by WHO as “The inability of a sexually active, non-contracepting couple to achieve pregnancy in one year” [1]. According to epidemiological data, it is estimated that an average of 10% of the global population in the reproductive age, are classified as infertile [2]. Male factor infertility has become a significant concern due to decreased quality of sperm, accounting for approximately 50% of infertility causes in the past decade [1].

A significant increase in the incidence of obesity in patients with male-factor infertility has been observed and couples with obese male partners are more likely to experience sub-fecundity [3]. Overweight in adult men has in recent years been associated with low semen quality [3–7], but some inconsistency still exists [8–10]. Adult male obesity has also been linked to sub-fertility, as measured by a prolonged waiting time to pregnancy [11–13].

More scholarly reports have documented that the decline in semen quality and male reproductive potential over the past half century has occurred relatively in parallel with increasing rates of obesity [14–16] suggesting it necessary to focus on the possibility of obesity as an etiology of male infertility and reduced fecundity.

Testosterone deficiency in obese men can possibly contribute to a decreases in semen quality [17], as measured by reduced total sperm count (TSC) [7] and a higher frequency of oligozoospermia [6] leading to a significantly negative relationship between BMI and total number of normal motile sperm [18]. Although changes in male reproductive hormones with increasing adiposity have been evident, the effects on sperm number and health remain unclear. A better understanding of the relationship between obesity and male fertility will allow physicians to better counsel men planning a family, about their body habitus [7,8,10,19–23].

Previous findings strongly suggest that gut microbiota contribute to complications related to high-fat diet feeding and metabolic disorders [24,25]. Moreover, the use of probiotics also mimics the key aspects of microbial symbiosis, enhancing reproductive fitness in mammalian hosts [26]. Different advantages of probiotics supplements have been previously reported, but studies on the effect of probiotic supplements on male fertility potential is still lacking.

This study assessed the possible effects of oral *Lactobacillus rhamnosus* PB01, DSM 14870 supplementation on the sperm quality parameters, reproductive hormone levels and lipid profiles in diet-induced obesity (DIO) male mice models.

Computer aided sperm analysis (CASA) systems can assess quantitative sperm parameters [27,28] with a highly improved and repeatable precision over manual methods which allow for the assessment and comparison of even small changes in sperm kinematics in response to the microenvironment [29]. To the best of the authors knowledge, this is the first study reporting the effects of probiotics on sperm kinematic parameters analyzed by CASA, which could be of interest for improving male fertility performance using probiotics supplementation.

## Materials and methods

Sperm progression (sperm categorized as immotile, non-progressively motile and progressively motile) was considered as the main outcome and the number of mice per group was calculated to accept a type 1 error of 0.05 and type 2 error of 0.10; comparable to previous similar studies [30,31]. Twenty-four, genetically outbred, 6-Week old male C57BL/6NTac mice (Taconic, Ejby, Denmark) were housed in a quiet room at 22°C to 24°C, with 60% relative humidity and 12-h dark-light cycles (light on from 0800 to 2000 h). Mice were allowed two weeks of adaptation and free access to their respective diets and tap water throughout the study. Handling of animals, used diets, housing, and animal experiments in this study were carried out in accordance with the “Guidelines for Animal Experimentation” and under approval of “The Danish Animal Experiments Inspectorate” no 2014-15-0201-00026.

### Study design

Phase I: After adaptation, the mice were randomly divided into two groups and fed with either a commercially available standard high fat diet (60%) (D12492, Research Diets Inc., USA) or a matched standard diet (ensuring correct ingestion of micronutrients and preventing micronutrient depletion) to produce the diet-induced obesity (DIO) and normal weight (NW) models, respectively.

**Fig 1:**
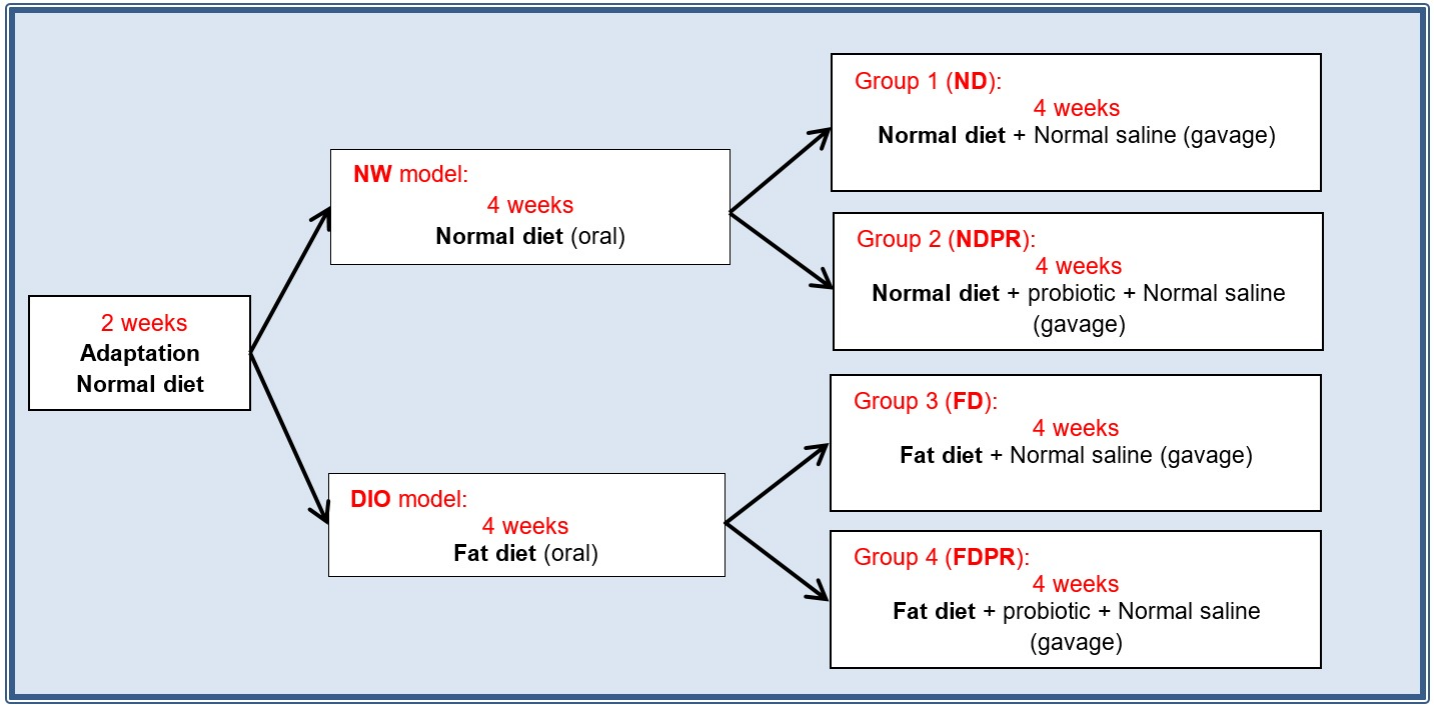
Study design and groups.

Phase II: The NW and DIO groups were randomly divided into two subgroups continuing the same standard or fat diet. However, one subgroup from each of the NW and DIO groups was randomly selected to receive the daily probiotic supplements diluted in normal saline by oral gavage. Because of the possible notorious stress caused by the gavaging process, the other subgroups also received oral gavage of normal saline with no probiotic supplements, resulting in the following four groups (Fig 1):

Group 1: Normal Diet (ND)
Group 2: Normal Diet + Probiotic supplement (NDPR)
Group 3: Fat diet (FD)
Group 4: Fat diet + Probiotic supplement (FDPR)

### Probiotics

*Lactobacillus rhamnosus* PB01, DSM 14870 was provided by Bifodan A/S (Hundested, Denmark) in the form of lyophilized powder. Based on the product information provided by the manufacturer (Bifodan A/S, Copenhagen, Denmark), the strain PB01 is genetically characterized employing two bio-molecular techniques. Species-level identification has been achieved by means of 16S rRNA gene sequencing, demonstrating that PB1 belonged to the species *Lactobacillus rhamnosus*. Pulsed Field Gel Electrophoresis (PFGE), employed for strain typing, enabled to obtain a strain specific macro-restriction pattern.

Aliquots providing 1×10^9^ colony- forming unit (CFU) per mouse were prepared (based on manufactures guidelines) and stored at -20°C until administration. Shortly before use, the prepared probiotic aliquots were diluted in normal saline as vehicle (0.25 ml), kept at room temperature and administered orally by a gavage needle to ascertain the presence of the probiotics in the gastrointestinal tract to both “NDPR” and “FDPR” groups. The “ND” and “FD” groups received oral administration of normal saline without probiotics. This process was performed once every day during phase II (weeks 4–8) of the study. The mice were weighted at week 0 (base), week 4 and week 8 of the study (to the nearest 0.01 g).

### Serum and tissue samples collection

At the end of the experimental period, the body weight of animals was measured and recorded. Blood samples were obtained from the facial vein of conscious mice and immediately centrifuged at 500g for 10 min at 4°C to collect blood serum. Serum samples were immediately stored and maintained at -20°C until analysis. The mice were euthanized by cervical dislocation before the testes and epididymides were collected by surgical dissection. After removing the epididymides the testes were weighted (to the nearest 0.001 g).

### Preparation of sperm suspensions

Sperm collection and preparation of suspension were carried out using a modified procedure described before [32,33]. Briefly, the caudal portion of the epididymis was placed in 30-mm petri dishes containing 2ml DMEM (Dulbecco's Modified Eagles Medium), cut open longitudinally and washed (by pipetting with the DMEM medium) to flush out all spermatozoa. The epididymis was removed, and the sperm supernatant fluid was allowed to incubated for 20 to 30 min at 37°C before being used for sperm analysis.

### ELISA assays

Blood serum was obtained by centrifugation (500 g for 10 min at 4°C) and stored at -20°C until being used to assess blood lipid profiles (total cholesterol (TC), high density lipoprotein (HDL), low/very low density lipoprotein (LDL/VLDL) and cholesterol) using a commercially available ELISA assay kit (ab65390, ABCAM, U.K.) according to the manufacturer’s directions.

The serum testosterone, FSH and LH levels were assessed using the total antioxidant capacity kit (ab65329, Abcam, United Kingdom), testosterone ELISA kit (ab108666, Abcam, United Kingdom), FSH ELISA kit (MBS703380, MyBioSource, U.S.A) and LH ELISA kit (MBS041300, MyBioSource, U.S.A), respectively (according to the manufacturers protocols).

### Sperm analysis

Sperm samples were assessed using the Sperm Class Analyzer (SCA^®^, version 5.4.0.0, Microptic S.L., Barcelona) computer-aided sperm analysis (CASA) system for sperm motility and kinematic parameters. The basic components of the Sperm Class Analyzer included a Nikon Eclipse 50i Upright Microscope (Nikon, Japan), a digital camera (Basler sca780-50, Basler, Germany) recording at 50 fps (frames per second), and a computer with the SCA® Software installed. The motility and concentration modules of the SCA have been rigorously evaluated and optimized to assess spermatozoa from several species including rodents [34,35].

#### Concentration, motility, and kinematic parameters

A Leja "chamber" slide (20 μm deep and 5 μl volume) (Leja Products B.V., Nieuw Vennep, Netherlands) was filled with five micro liters of the sperm suspension and placed onto a temperature controlled stage (37°C) of the Nikon E50i microscope. A 10× negative phase contrast objective in conjunction with a phase contrast condenser was used to study sperm motility using the motility module of the SCA.

The SCA® concentration and motility module provided the sperm concentration, detailed velocity, and motion-path kinematic parameters (Table 1). The total sperm count was calculated based on the concentration (Mil/ml) and total volume of the prepared sperm suspension (2 ml). The SCA default cut-off values for fast, medium, and slow swimming spermatozoa were based on curvilinear velocity (VCL) = Fast > 45 > Medium > 35 > Slow.

**Table 1:** Sperm kinematic parameters defined by the SCA CASA system [36].

Parameter	Unit	Description of the Parameter
**Motility**	%	Percentage of sperm in different motility groups based velocity and progression
**Concentration**	×10^6^ mL^-1^	Number of spermatozoa per milliliter
**VCL**	Μm s^-1^	Curvilinear velocity along actual swimming path
**VSL**	μm s^-1^	Straight-line velocity along shortest path from start to end point
**VAP**	μm s^-1^	Average path velocity based on every 11^th^ frame of VCL path
**LIN**	%	Linearity of a curvilinear path, expressed as VSL/VCL
**STR**	%	Straightness, expressed as VSL/VAP
**WOB**	%	Wobble, expressed as VAP/VCL
**ALH**	Μm	Amplitude of lateral head displacement
**BCF**	Hz	Beat cross frequency based on VCL crossing VAP per second

### Statistical analysis

Data are shown as means ± standard deviation (SD). One-way repeated measures analysis of variance (ANOVA) was used to compare differences in weight between groups and the time points analyzed for, total sperm counts, motility, lipid profile parameters (TC, HDL, and LDL/VLDL) and hormone levels (LH, FSH and Testosterone); Bonferroni post-hoc test was used for pair-wise comparison wherever ANOVA yielded a statistical significant difference. Independent samples t-test was used to compare the sperm kinematic parameters between control and probiotic treated sub-groups in NW (ND vs. NDPR) and DIO (FD vs. FDPR) groups separately. A p-value less than 0.05 (P<0.05) was considered significant unless otherwise stated. The “IBM^®^ SPSS^®^ Statistics” version 23 (SPSS Inc., Chicago, IL, USA) was used to perform statistical analysis.

## Results

### Probiotics supplementation effects on testis and total body weight

The diet-induced obesity (DIO) groups being fed on fat diet, demonstrated significantly higher (P<0.01) body weight than the normal weight group (on normal diet) at week four (Table 2).

**Table 2:** Estimated marginal means of body weight in mice fed normal or high fat diet at weeks 0 and 4, followed by estimated marginal means of body weight, testicular weight, testis to body weight ratio; and total sperm count (Median (25–75 percentiles)) in groups fed on normal diet (ND), high fat diet (FD), normal diet with probiotics (NDPR) or fat diet with probiotics (FDPR) during the second 4 weeks (week 4–8). Data are presented as means ±SD. Similar letters demonstrate significant differences between different groups in week 4 and week 8 (P<0.05).

	Week 0 (base)	Week 4	Week 8				
Diet	Body weight	Body weight	Diet	Body weight	Testicular weight	Testis/Body Weight	Total sperm count × 10^6^
				(g ± SD)	(g ± SD)	ratio	Median (25–75 percentiles)
Normal diet	21.42 ± 1.24	25.42 ± 1.38 ^A^	NDPR	26.00 ± 1.10 ^C,D^	0.102 ± 0.021 ^H,J^	0.004 ± 0.0008 ^L^	52.9 (46.2, 59.6)
			ND	27.83 ± 1.17 ^E,F^	0.107 ± 0.010 ^G,K^	0.004 ± 0.0005 ^M^	62.1 (51.4, 71.2)
Fat diet	21.75 ± 2.01	32.67 ± 1.97 ^A^	FDPR	34.50 ± 1.97 ^B,C,E^	0.127 ± 0.005 ^G,H,I^	0.004 ± 0.0004 ^N^	56.4 (46.4, 69.8)
			FD	38.67 ± 2.07 ^B,D,F^	0.117 ± 0.005 ^I,J,K^	0.003 ± 0.0002 ^L,M,N^	44.7 (36.0, 50.8)

From week four to week eight, groups on normal and fat diet without probiotic supplementation (ND and FD) demonstrated increased body weight (P = 0.185 and P≤0.001 respectively), while the probiotic supplemented groups on the same diets (NDPR and FDPR) maintained a stable weight with no significant change (Table 2).

The rising trend of the mean body weight in the FD group as opposed to the stable weight of the FDPR group resulted in a significant difference at the end of week 8 (P≤0.01), while the weight gain in the ND group did not reach a significant difference compared to the NDPR group (Table 2).

### Testicular weight

Probiotic supplementation showed no significant effect (P = 0.903) on the mean testicular weight between groups fed on normal diet (ND and NDPR). However, DIO groups with or without probiotic supplementation (FD and FDPR), both demonstrated significantly higher (P≤0.01) mean testicular weights compared to the ND and NDPR groups. The probiotic supplemented DIO group also had a higher (P = 0.034) mean testicular weight compared to the DIO group without probiotic supplementation. The testis to body weight ratio in the DIO group without probiotic supplementation (FD) was significantly (P≤0.05) lower than all other groups (ND, NDPR and FDPR) (Table 2).

### Probiotics supplementation effects on total sperm counts and sperm motility (Progression)

The DIO group without probiotic supplementation (FD) demonstrated a notably lower mean total cell count compared to the probiotic supplemented DIO group (FDPR) and the control groups (ND and NDPR) but the differences were not significant between any of the test and control groups (Table 2).

**Fig 2:**
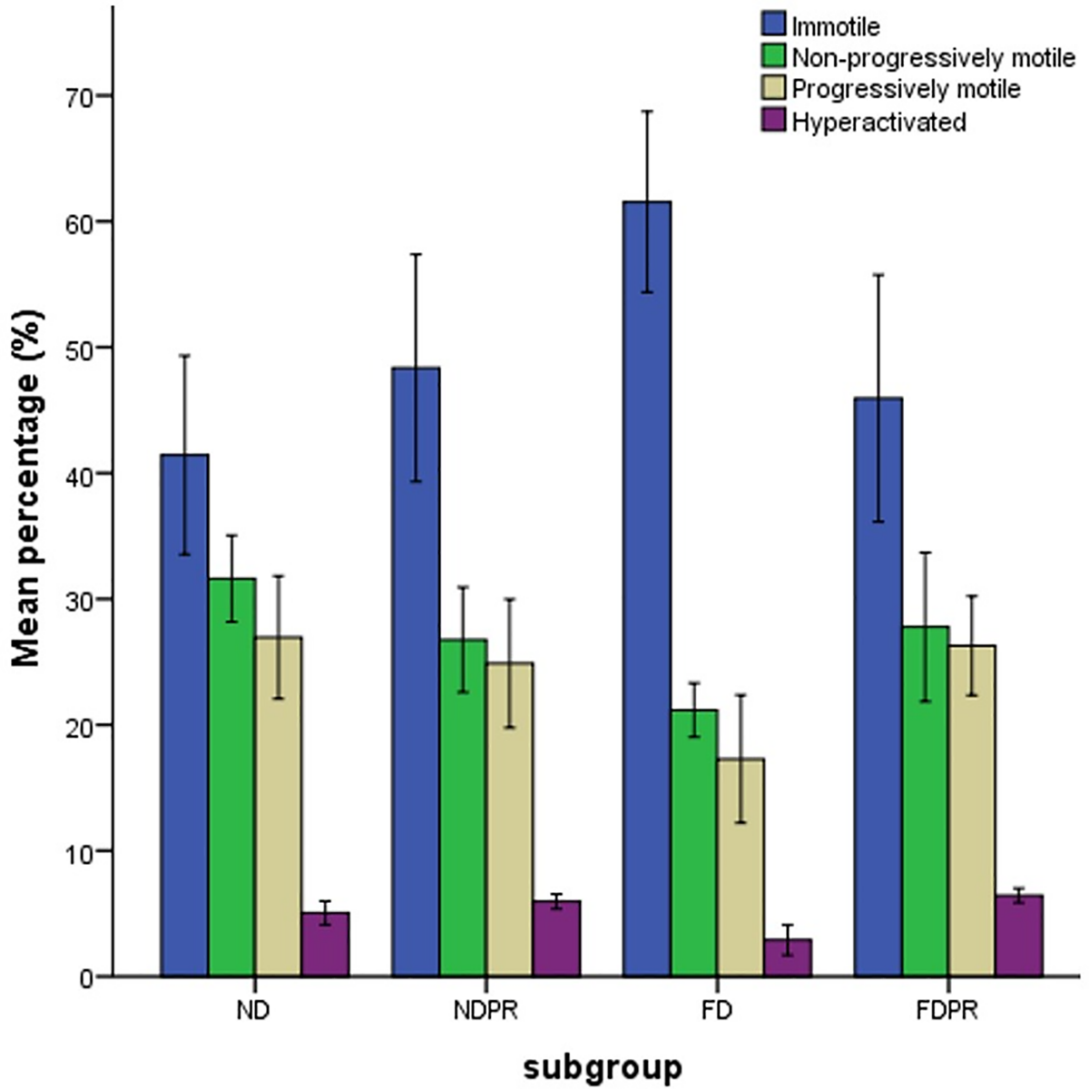
The estimated marginal means of percentage of immotile, non-progressively motile, progressively motile and hyperactivated (% of motile) sperm in groups fed on normal diet (ND), high fat diet (FD), normal diet with probiotic supplementation (NDPR) and fat diet with probiotic supplementation (FDPR). Data are presented as means and bars indicate SD. Similar letters demonstrate significant difference (P < .05).

The groups on normal diet with or without probiotic supplementation (ND and NDPR) demonstrated similar percentages of sperm in different categories of progressiveness (Fig 2). However, the FD group had a significantly lower percentage (P≤0.01) of non-progressively and progressively motile sperm compared to the ND group (Fig 2).

The probiotic supplemented fat-diet group (FDPR) also demonstrated a significantly lower percentage of immotile sperm (P≤0.01) and significantly higher percentage of non-progressively (P≤0.05) and progressively motile (P≤0.01) sperm compared to the FD group (FD) (Fig 2).

The FD group also demonstrated a significantly lower (P≤0.01) percentage of hyperactivated sperm compared to the normal diet (ND) and probiotic supplemented fat- and normal- diet groups (FDPR and NDPR) (Fig 2).

### Probiotics supplementation effects on kinematic parameters

**Fig 3:**
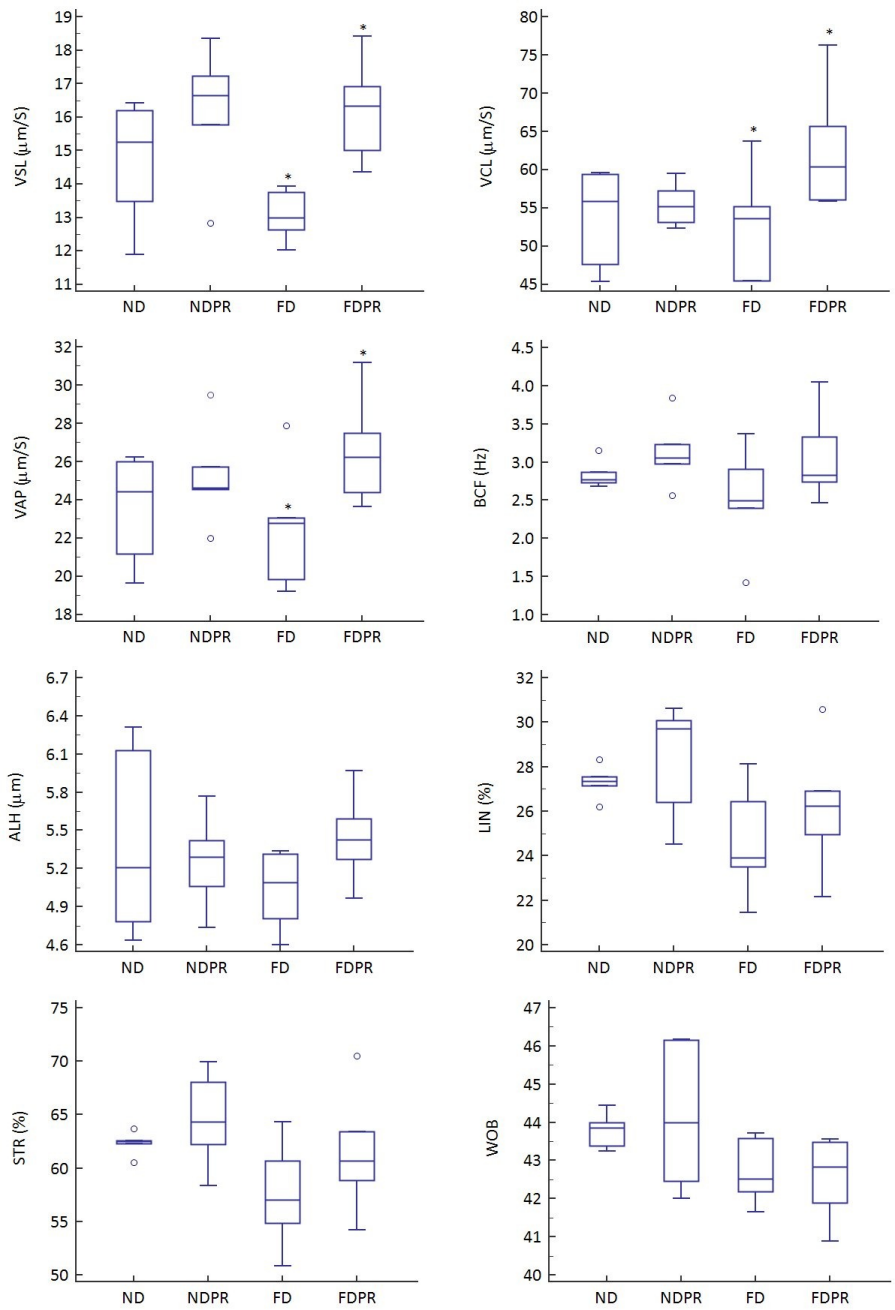
Box and whisker plots demonstrating the total average sperm kinematic parameters (Curvilinear velocity (VCL), Average path velocity (VAP), Straight-line velocity (VSL), Beat cross frequency (BCF), Amplitude of lateral head displacement (ALH), Linearity of a curvilinear path (LIN), Straightness (STR), Wobble (WOB) in mice fed normal diet (ND), high fat diet (FD), normal diet with probiotics (NDPR). * demonstrate significant pairwise differences (P<0.05).

All the kinematic parameters in the FD group demonstrated lower mean values compared to the ND group (Fig 3). The VCL, VAP, VSL, BCF and ALH parameters demonstrated insignificant higher mean values in the probiotic supplemented high fat diet and normal diet groups (NDPR and FDPR) compared to the groups without probiotic supplementation (ND and FD). However, this difference was significant for VSL (P<0.01), VAP (P<0.05) and VCL (P<0.05) between the FD and FDPR groups (Fig 3).

The detailed sperm kinematic parameters in groups fed on normal diet with or without probiotic supplementation (NDPR and ND) did not show a significant difference when categorizing sperm based on slow, medium, and rapid velocity. However, the probiotic supplemented groups on fat diet (FDPR) had a significantly higher VCL, VSL, VAP and ALH in the Rapid category, and significantly higher VCL in the Slow category (Table 3).

**Table 3:** Marginal means and pairwise differences of detailed kinematic parameters within the slow, medium, and rapid velocity categories in mice on normal diet (ND), fat diet (FD), ND with probiotics (NDPR) and FD with probiotics (FDPR). Provided values are Mean ± SEM. (* demonstrates pairwise significant differences (P < 0.05)).

	Normal weight			Diet induced obesity		
	Control	Probiotic supplement	Pairwise difference	Control	Probiotic supplement	Pairwise difference
	(ND)	(NDPR)	(Mean ± SEM)	(FD)	(FDPR)	(Mean ± SEM)
**VCL**						
**Slow**	12.29 ± 0.08	12.28 ± 0.08	0.01 ± 0.05	12.05 ± 0.16	12.27 ± 0.13	-0.22 ± 0.09 *
**Medium**	25.41 ± 0.45	26.01 ± 0.80	-0.60 ± 0.38	26.36 ± 0.82	26.32 ± 1.03	0.04 ± 0.54
**Rapid**	107.60 ± 6.71	107.98 ± 7.68	-0.38 ± 4.17	95.18 ± 7.54	110.7 ± 4.73	-15.52 ± 3.63 *
**VSL**						
**Slow**	2.13 ± 0.25	2.10 ± 0.46	0.02 ± 0.21	1.80 ± 0.25	2.06 ± 0.32	-0.27 ± 0.17
**Medium**	5.26 ± 0.34	5.43 ± 0.36	-0.17 ± 0.20	5.72 ± 0.62	5.58 ± 0.29	0.14 ± 0.28
**Rapid**	31.91 ± 1.63	33.45 ± 4.77	-1.55 ± 2.06	24.82 ± 4.12	30.75 ± 5.07	-5.94 ± 2.67 *
**VAP**						
**Slow**	4.46 ± 0.33	4.17 ± 0.69	0.28 ± 0.31	3.88 ± 0.42	4.19 ± 0.30	-0.31 ± 0.21
**Medium**	10.59 ± 0.32	10.68 ± 0.75	-0.09 ± 0.33	11.25 ± 0.55	10.73 ± 0.71	0.52 ± 0.36
**Rapid**	48.26 ± 2.84	49.07 ± 4.03	-0.81 ± 2.01	41.64 ± 4.01	48.09 ± 2.90	-6.45 ± 2.02 *
**LIN**						
**Slow**	17.29 ± 2.02	17.13 ± 3.70	0.17 ± 1.72	14.88 ± 1.88	16.80 ± 2.61	-1.92 ± 1.31
**Medium**	20.68 ± 1.06	20.90 ± 1.63	-0.22 ± 0.79	21.74 ± 2.76	21.18 ± 0.57	0.56 ± 1.15
**Rapid**	29.68 ± 0.68	30.88 ± 2.92	-1.21 ± 1.22	26.00 ± 2.87	27.70 ± 3.64	-1.70 ± 1.89
**STR**						
**Slow**	47.60 ± 2.38	50.3 ± 5.78	-2.70 ± 2.55	46.22 ± 2.60	49.10 ± 6.72	-2.88 ± 2.94
**Medium**	49.61 ± 2.21	50.89 ± 1.94	-1.28 ± 1.20	50.90 ± 5.85	52.03 ± 1.38	-1.13 ± 2.45
**Rapid**	66.15 ± 1.06	67.90 ± 4.79	-1.74 ± 2.00	59.39 ± 5.25	63.62 ± 6.55	-4.23 ± 3.43
**WOB**						
**Slow**	36.25 ± 2.64	33.97 ± 5.55	2.28 ± 2.51	32.17 ± 3.19	34.17 ± 2.29	-2.00 ± 1.60
**Medium**	41.67 ± 0.59	41.07 ± 2.75	0.60 ± 1.15	42.70 ± 1.80	40.74 ± 1.61	1.96 ± 0.98
**Rapid**	44.86 ± 0.34	45.43 ± 1.71	-0.58 ± 0.71	43.71 ± 1.40	43.43 ± 1.57	0.28 ± 0.86
**ALH**						
**Medium**	2.38 ± 0.19	2.34 ± 0.10	0.05 ± 0.09	2.40 ± 0.25	2.34 ± 0.11	0.07 ± 0.11
**Rapid**	7.35 ± 0.51	6.92 ± 0.29	0.42 ± 0.24	6.58 ± 0.42	7.16 ± 0.20	-0.57 ± 0.19 *
**BCF**						
**Medium**	1.28 ± 0.09	1.18 ± 0.14	0.10 ± 0.07	1.29 ± 0.65	1.15 ± 0.22	0.14 ± 0.28
**Rapid**	3.86 ± 0.33	4.20 ± 0.57	-0.34 ± 0.27	3.38 ± 0.61	4.51 ± 1.03	-1.13 ± 0.49

### Probiotics supplementation effects on lipid profiles and reproductive hormones

The results of the reproductive hormones and lipid profiles in mice fed a normal diet (ND), high fat diet (FD), normal diet with probiotics (NDPR) and fat diet with probiotics (FDPR) at the end of the second phase of the study can be seen in Table 4.

**Table 4:** Reproductive hormones, lipid profiles and total antioxidant capacity (TAC) in mice on normal diet (ND), fat diet (FD), ND with probiotics (NDPR) and FD with probiotics (FDPR). Provided values are mean ± standard deviation. (Superscripted letters mark pairwise significant differences (P < 0.05)).

	ND	NDPR	FD	FDPR
LH (mIU/ml)	2.13 ± 0.39	2.17 ± 0.22	2.03 ± 0.24	2.21 ± 0.16
FSH (mIU/ml)	7.22 ± 1.73	7.72 ± 2.05	6.36 ± 0.52	9.84 ± 2.38
Testosterone (ng/ml)	1.63 ±0.30	1.96 ± 0.09 ^**a**^	1.20 ± 0.42 ^**a**^	1.50 ± 0.40
LDL/VLDL (μg/μl)	0.18 ± 0.02 ^**a**^	0.14 ± 0.02 ^**b**^	0.32 ± 0.05 ^**a,b,c**^	0.21 ± 0.03 ^**c**^
HDL (μg/μl)	0.66 ± 0.16 ^**a**^	0.56 ± 0.05 ^**b,c**^	1.42 ± 0.24 ^**a,b**^	1.46 ± 0.46 ^**c**^
Total Cholesterol (μg/μl)	1.05 ±0.15 ^**a,b**^	0.96 ± 0.09 ^c,d^	1.73 ± 0.01 ^**a,c,e**^	1.56 ± 0.07 ^**b,d,e**^
TAC (total antioxidant capacity, ng/ml)	34.069±1.54 ^**a**^	37.86±1.95 ^**a,b,c**^	25.88±8.90 ^**c**^	31.26±3.39 ^**b**^

LH and FSH hormone levels were similar in groups on normal diet with and without probiotic supplementation (NDPR and ND) while the DIO groups with and without probiotic supplementation (FDPR and FD) demonstrated higher LH and lower FSH serum hormone levels than the control groups (ND, NDPR), however insignificant.

Testosterone levels in the DIO groups were generally lower than the groups on normal diet while this difference was only significant (P≤0.05) between the FD and NDPR groups.

The groups on normal diet (ND and NDPR) showed generally lower HDL, LDL/VLDL and total cholesterol levels than the groups on fat diet (FD and FDPR). However, the FD group showed significantly higher (P≤0.01) levels of HDL, LDL/VLDL and total cholesterol than the ND and NDPR groups. The LDL/VLDL and total cholesterol levels in the FD group was also significantly higher (P≤0.01) than the FDPR group. The FDPR group also demonstrated significantly higher (P≤0.01) values of total cholesterol compared to the ND and NDPR groups.

The groups on fat diet had lower total antioxidant capacity (TAC) levels than the groups on normal diet while the NDPR group demonstrated significantly higher (P≤0.05) (TAC) levels compared to all other groups.

## Discussion

The nociceptive and weight regulatory effect of *L*. *rhamnosus* PB01 oral supplementation in diet-induced obesity mice models [37] has been previously documented. The same strain from Bifodan A/S (Hundested, Denmark) was used for the current study with the hypothesis that the proven weight lowering effects of *Lactobacillus rhamnosus* PB01, DSM 14870 [37] could also exert a positive effect on reproductive hormones and sperm quality.

The significant weight loss seen after four weeks of *L*. *rhamnosus* (PB01, DSM 14870) supplementation in the DIO group, is in compliance with the previous findings on the weight lowering effects of other probiotics [38–40]. The weight lowering effect of the *Lactobacillus* strains has been previously explained by several mechanisms in the literature [24,25,41–45]. However, the observed significantly lower weight of the normal diet group after only four weeks of probiotic supplementation (at Week 8) compared to the normal diet group without probiotic supplementation, may be suggestive of the possibility of probiotics decreasing the normally required absorption of lipids, and possibly other nutrients, thus preventing normal age-dependent weight gain which may be considered as an adverse effect in non-obese cases; however, this hypothesis requires further investigation.

The higher testicular weight observed in the DIO compared to the NW groups could be due to the higher sucrose levels provided in the high fat diet [46]. Additionally, the significantly higher testicular weight in probiotic supplemented DIO mice in comparison to their age- and diet-matched controls with no probiotic supplementation, could be related to the higher levels of circulating testosterone and prevention of diet-related testicular atrophy by the probiotic supplementation as suggested by previous studies [30]. Other *Lactobacillus rhamnosus* strains *(L*. *rhamnosus GG*) have also shown significantly increased seminiferous tubule cross-sectional profiles, and increased spermatogenesis and Leydig cell numbers per testis in mice [30].

The increased levels of serum TC, LDL, and HDL in the high-fat diet groups were similar to previous studies using the same high fat diet (D12492, Research Diets Inc., USA) [47–49]. The lower levels of total antioxidant capacity (TAC) in the high-fat diet group, were also in agreement with previous studies demonstrating a reduced antioxidant capacity as a result of obesity [50,51]. Hence, it confirmed that the high fat diet resulted in a hyperlipidemic murine model potentially producing the adverse effects of hyperlipidemia on male fertility. The lower total cholesterol and LDL/VLDL levels in both NW and DIO probiotic supplemented groups compared to their respective control groups may be associated with the hypolipidemic activity of probiotics as reported in previous studies [52].

It has also been shown that high-fat diet can provoke oxidative stress and consequently sperm damage while probiotic supplementation reduces the level of oxidative damage and improves sperm quality to some extent [53].

### Effect of probiotics on male reproductive hormones

The lower level of testosterone in the DIO groups in this study is in compliance with the previous reports of decreased serum testosterone levels in obese and hyperlipidemic mice [54,55] and humans [8,10,17]; possibly due to the degenerative and detrimental effects of cholesterol-rich diets on the secretory capacity of Leydig and Sertoli cells [56].

The lower levels of LH and FSH in the DIO group can be associated with the conversion of androgens to estradiol, resulting in raised serum estrogen levels [6,57]. The increased estradiol levels in DIO models would reduce the production and secretion of FSH and LH, resulting in reduced testicular function and testosterone production (both intra-testicular and circulating Testosterone) [6,58]. Excessive estradiol has also been suggested to have a direct toxic effect on spermatogenesis [6,58].

Both DIO and NW groups supplemented with probiotics in this study demonstrated higher testosterone, LH and FSH levels after 4 weeks of probiotic supplementation. Previous studies have also reported improved levels of FSH, LH and testosterone levels in humans [59] and higher levels of circulating testosterone in mice [30] supplemented with other strains of *Lactobacillus* (*L*. *paracasei B21060* and *L*. *reuteri* respectively), regardless of the type of diet.

### Effect of probiotics on sperm count, motility, and kinematics

In this study, increased weight gain resulted in reduced percentage of progressively and non-progressively motile sperm. Previous studies have also demonstrated that obesity can impair male fertility not only by reducing sperm quality [6,23], but also by altering the spermatogenesis process and affecting sperm maturation [6,57].

The extremely high viscosity of the cauda epidydimal fluid which is mainly due to extremely high sperm numbers, renders it difficult to accurately aspirate a specific sperm volume from the tiny cauda epidydimal tubule; hence the large variation of results due to the used sampling protocol (despite being the current preferred method in most studies) may produce inaccurate assessments of the total sperm count (TSC) which should therefore be interpreted cautiously. TSC has been previously associated with testosterone levels during spermatogenesis and maturation [60,61]. The control groups (ND and NDPR) and the probiotic supplemented DIO group (FDPR) demonstrated similar testosterone and accordingly similar TSC levels. Although insignificant, the lower TSC in the DIO group without probiotic supplementation (FD) compared to the probiotic supplemented DIO group (FDPR), may be related to the lower level of testosterone in this group.

Sperm motility has been identified as the most important feature of the spermatozoon reflecting on several functional and structural competencies, including spermatozoa metabolism [62] and is considered as an important indicator of sperm function. The new generations of computer aided sperm analysis (CASA) systems can provide more accurate, objective and reproducible results for various motility parameters, not possible using conventional manual methods [63].

The significantly lower percentage of non-progressively and progressively motile sperm and significantly higher percentage of immotile sperm in the fat diet group (FD) compared to the normal diet group (ND) can confirm the negative effects of obesity on sperm motility as also reported by several previous studies on rodents and other species [18,35,64–67].

The demonstrated significantly lower percentage of immotile (static) and significantly higher percentage of progressively motile sperm in the probiotic supplemented fat-diet group (FDPR) compared to the fat diet group (FD) also supports the results of previous studies assessing the effect of probiotics on rodents [14,53] and humans [59].

Sperm motility is acquired while sperm are transiting through the epididymal duct [68,69]. The epididymal structure and functions are dependent on the presence of androgen [70] and more specifically dihydrotestosterone [71] which is produced by conversion of testosterone catalyzed by “5α-Reductase (types I and II)” enzymes [71]. Thus, the reduction in sperm motility in the DIO groups in this study may be due to the decrease in serum testosterone (and consequently dihydrotestosterone) alongside the increasing body weight. The weight reduction and hormonal regulating effect of the probiotics could be associated with the better quality of the sperm motility in the probiotic supplemented groups associated with the above-mentioned changes. However, an exact mechanism is yet to be identified.

The precision and accuracy of CASA systems make it possible to detect subtle changes in sperm motion and kinematic parameters as useful markers for assessing toxicity [35]. Different sperm motility and kinematic parameters can be related to specific functions; sperm progression can be indicated by VSL, VAP, STR and LIN while VCL, ALH and BCF are pointers of sperm vigor [35]. LIN and STR describe the swimming pattern, while VCL and BCF can be an indication of sperm viability [72,73].

The DIO mice demonstrated lower values in all kinematic parameters, also observed as the reduction in the level of progressive motility, possibly implying that spermatozoa will not be able to migrate along the female reproductive tract efficiently enough and reach the site of fertilization in-vivo [35].

The increased motion path parameters including Linearity (Lin), Straightness (STR) and beat cross frequency (BCF) and significantly (P<0.05) higher velocity parameters (VSL, VCL and VAP) in the probiotic supplemented DIO group compared to its respective control without probiotic supplementation suggests a general capability of probiotics in reversing the deleterious effects of DIO on sperm swimming speed.

The motion path parameters including Linearity (Lin), Straightness (STR) and beat cross frequency (BCF) were also increased in both DIO and NW groups after 4 weeks of probiotic supplementation while ALH was marginally-significantly (P = 0.057) higher in the FDPR compare to the FD group.

The significantly higher VSL, VCL, VAP and ALH of sperm in the “rapid velocity” category in the probiotic supplemented DIO groups may be interpreted as the increased capability of the sperm to reach the oocyte during its migration in the female reproductive tract and further emphasizes the positive effect of probiotic supplementation on the kinematic parameters of the sperm.

The probiotics’ mechanism of action in reversing the adverse effects of DIO on sperm kinematic parameters has not been studied in detail yet, but could be associated with the increasing level of testosterone during spermatogenesis and maturation, reducing the level of oxidative damage and inducing weight loss, which would also possibly reduce scrotal fat regulating the testes temperature. Furthermore, recent studies of diet and exercise interventions in an obese mouse model [74] demonstrated that the metabolic health of an individual is correlated with sperm function. Improvements in metabolic health such as the return of cholesterol to normal levels result in better sperm motility associated with improvements to molecular composition such as reductions in oxidative stress and reduced DNA damage model [74]. Further investigation is still required to determine the exact mechanism of action for the protective effect of probiotics on the sperm kinematic parameters. Based on the impact of four weeks of probiotic consumption on weight and lipid profiles seen in this study, it can be proposed that *Lactobacillus rhamnosus* PB01 could act as a potential weight and lipid profile regulator. Hence, it could be suggested that the mentioned probiotic might also deliver a positive influence on hormonal balance, sperm production and ultimately sperm quality associated with the above-mentioned changes.

## Conclusion

“This study demonstrated that Lactobacillus rhamnosus PB01 (DSM 14870) could affect weight and also some male fertility potential biomarkers including sperm motility parameters and hormones in diet-induced obesity model. The change in the sperm velocity and motion path parameters demonstrated in this study, may be associated with the direct effect of probiotics on spermatogenesis and maturation process or indirectly by removing the adverse effects of obesity and increasing the level of total antioxidant capacity. The hormonal balance induced by the probiotics could also be playing a positive role in the improvement of the sperm motility parameters. However, considering the value of these findings, larger studies accounting for the possible variability in sperm kinematics and/or hormonal influence, further investigating the underlying mechanisms of the positive effect of probiotics on male fertility potential under obesity conditions are required to provide a more solid conclusion.
